# Data on the activity of place cells in the hippocampal CA1 subfield of a monkey performing a shuttling task

**DOI:** 10.1016/j.dib.2019.104467

**Published:** 2019-09-01

**Authors:** Yutaro Hazama, Ryoi Tamura

**Affiliations:** Department of Integrative Neuroscience, Graduate School of Medicine and Pharmaceutical Sciences, University of Toyama, 2630 Sugitani, Toyama 930-0194, Japan

**Keywords:** Hippocampus, Macaques, Unit recording, Place cells, Freely behaving condition, Spatial cognition

## Abstract

This data article provides spike-timestamps of place cells recorded in a male Japanese monkey and the animal's sequential positions during the performance of a shuttling task on a rectangular track. All data were recorded in the right hippocampal CA1 subfield, while the monkey performed the task under a freely behaving condition. These were the source data on the monkey place cells in our related research article entitled “Effects of self-locomotion on the activity of place cells in the hippocampus of a freely behaving monkey” [1]. In addition, here we show a movement directional activity of a place cell in two-dimensional space as an example of data utilization. The source data are freely accessible [2] and can be used by other researchers to obtain new insights into place cells, such as functional differences between animal species.

**Table:** Specifications Table

Subject area	*Neuroscience*
More specific subject area	*spatial navigation, hippocampus, place cells*
Type of data	*MAT file (.mat), figure*
How data was acquired	*Electrical signals (neuronal activity) from a tetrode, fed to a unity-gain pre-amplifier, were amplified by a main amplifier (Lynx-8, Neuralynx); the signals were AD-converted via a multi-function board (DT3010, Data Translation) and stored in a hard disk. A CCD camera recorded the image containing the behaving animal; the images were stored on the hard disk through a frame grabber board (DT3162, Data Translation). These data were analyzed using MATLAB.*
Data format	*Raw and analyzed*
Experimental factors	*Data from one male Japanese monkey (Macaca fuscata) were used in this article. Hippocampal pyramidal cell activity was recorded with a tetrode.*
Experimental features	*Extracellular recording in the hippocampal CA1 subfield of a freely behaving monkey*
Data source location	*Department of Integrative Neuroscience, Graduate School of Medicine and Pharmaceutical Sciences, University of Toyama, 2630 Sugitani, Toyama 930-0194, Japan*
Data accessibility	*The data stored in our university repository**http://hdl.handle.net/10110/00019073*[2]*are freely accessible.*
Related research article	*Y. Hazama, R. Tamura, Effects of self-locomotion on the activity of place cells in the hippocampus of a freely behaving monkey, Neurosci. Lett. 2019*[1].

**Table:** 

**Value of the Data** • The spike-timestamp data and the animal's position data can be processed by any researcher. • Movement direction could modulate the monkey place cell activity in two-dimensional space. • The present data can be used to compare the findings of place cells between the monkey and other animal species.

## 1 Data

**Fig. 1:**
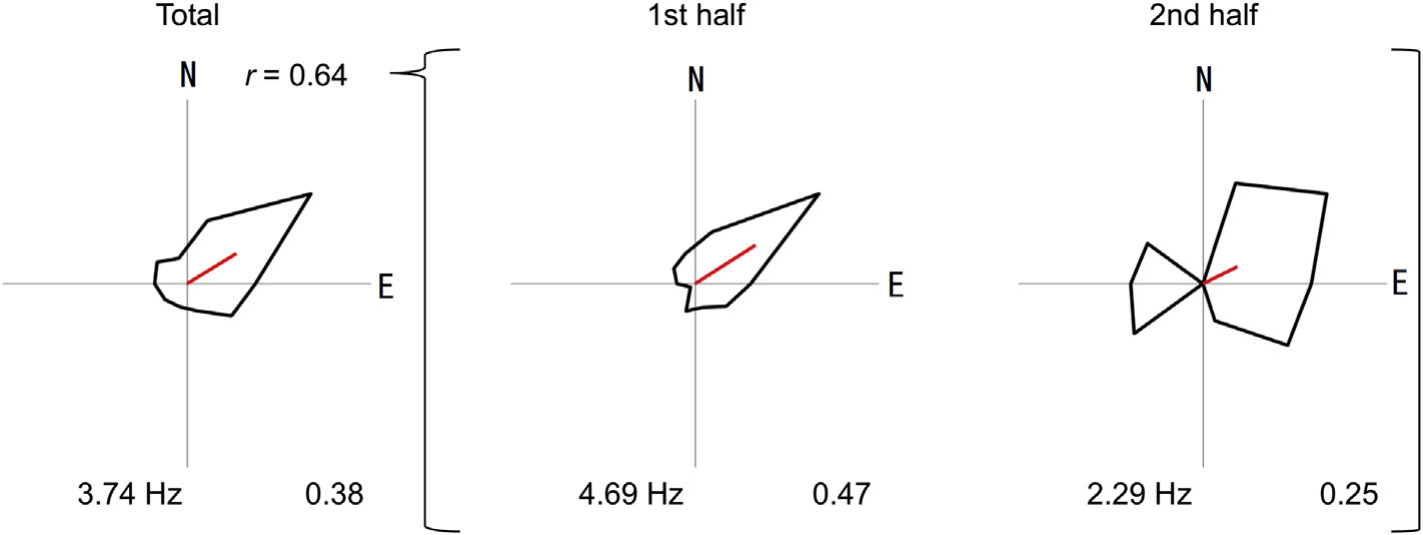
**Movement directional activity of a place cell.** Circular distribution of firing rates during whole recording trial (left), the 1st half of the trial (middle) and the 2nd half of the trial (right). The x- and y-axes in the polar coordinates system are parallel to the long and minor axes of the track, and each positive direction of the x- and y-axes means east (E) and north (N) directions. Peak firing rate is presented at lower left. The *r* value between the maps indicates the Pearson correlation coefficient between circular distributions of firing rates in the 1st and 2nd half of a single trial. Rayleigh vector length of the circular distribution is presented at lower right. This neuron consistently showed significant tuning of movement direction, i.e., the cell increased firing when the monkey moved in the east-northeast direction both in the 1st and 2nd half of a task trial.

The present data set contains spike-timestamps of 18 place cells recorded in the right hippocampal CA1 subfield of a male Japanese monkey performing a shuttling task on a rectangular (4 × 0.9 m) track, and his sequential positions on it. The monkey was monitored by a CCD camera every 33.3 ms, and his position as a pixel (a pixel size: 0.9 × 0.9 cm) on the track was determined from the image. These data are freely accessible [2]. Each spike-timestamp file has one column showing the timestamp (in ms) when the place cell fired during the task (time 0 corresponds to the onset of a task trial). Each position file has three columns: the sequential frame numbers of the CCD camera images are shown in the first column, the x-positions (1 and 440 correspond to the west-end and east-end pixels, respectively, of the track) in the second column, and the y-positions (1 and 99 correspond to the south-end and north-end pixels, respectively, of the track) in the third column. As an example of data utilization, we show a newly analyzed result, i.e., movement directional activity of a place cell in two-dimensional space, (spike-timestamp file: MonkeyPosition_ID13.mat, position file: SpikeTimeStamp_ID13.mat) in Fig. 1.

## 2 Experimental design, materials, and methods

### 2.1 Subject

One male Japanese monkey (*Macaca fuscata*) was used. The monkey was treated in strict compliance with the Animal Care and Use Committee of University of Toyama, and with the NIH Guide for the Care and Use of Laboratory Animals. The protocol was approved by the University of Toyama's ethics committee for Animal Care and Use (protocol approval #: A2016MED-36).

### 2.2 Behavioral task

A linear track was used for a behavioral task. A pellet dispenser was set at both ends of the track. The monkey was required to shuttle back and forth on the track to get rewards (small pieces of pellets) alternatively delivered by the two dispensers. A task trial consisted of 17 shuttle movements.

### 2.3 Implantation of tetrode for unit recording

Before the implantation of tetrode, a cranioplastic cap was surgically attached to the monkey skull, and depth profiles of evoked local field potentials were recorded in the hippocampus to establish precise coordinates of CA1 pyramidal cell layer. The tetrode consisting of four-twisted 25-μm polyvinyl formal-coated nichrome wires was implanted through a stainless-steel guide cannula, targeting the area located just above the hippocampus. A handmade position adjustment device was fixed to the cranioplastic cap, and the tetrode was fixed to it in order to fine-tune the depth of the electrode tip in the CA1 area.

### 2.4 Data acquisition and storage in unit recording sessions

Electrical signals from the tetrode, fed to a unity-gain pre-amplifier, were amplified 9000 times with band-pass filtering of 300-9000 Hz. When clear and stable neuronal activities were observed, we started the unit recording session. Before the start of a task trial the monkey was placed in a seated position on a chair having a front door. A few seconds before the trial start, two light bulbs fixed at the monkey's head cap were switched on, and a transistor-transistor logic (TTL) pulse triggered data acquisition (neuronal signals and CCD camera images). At the beginning of a trial the front door of the chair was opened, giving the monkey free access to the track (and the initiation of shuttling). When the monkey finished a trial (17 shuttle movements), he returned to the chair; the light bulbs were switched off and data acquisition for that trial was manually stopped (image capturing first, neuronal data next). A few minutes were allowed to elapse before the next trial.

Neuronal signals were digitized at 50 kHz by a multi-function board (DT3010, Data Translation) and stored on a computer hard disk for offline analysis; the CCD camera, set at the ceiling of the experimental room and directed vertically downwards to the linear track, recorded the image of the track containing the animal at a rate of 30 frames/s; the images were stored on the hard disk through a frame grabber board (DT3162, Data Translation). For the image data, after removing lens distortion using the camera calibration toolbox in MATLAB, the image of the linear track was expressed in 440 × 99 pixels (1 pixel = ca. 0.9 × 0.9 cm). The center of gravity of the light bulbs (detected as high signal intensity areas) in each frame was calculated to estimate the monkey's horizontal (x-y) position in the calibrated image.

### 2.5 Spike sorting and classification of neuronal types

The waveforms of spikes (0.5 ms-pre and 1.5 ms-post spike onset) that crossed an experimenter-defined threshold were extracted from the signals and stored on the hard disk with a timestamp. The features of the spike waveform (spike energy, spike amplitude, spike width and principal components) were used for sorting with MClust-3.5 (A. D. Redish and others, University of Minnesota, USA, http://redishlab.neuroscience.umn.edu/MClust/MClust.html). Spike sorting was performed automatically using KlustaKwik (K. Harris, Rutgers University, USA, http://klusta.readthedocs.io/en/latest/). The isolated clusters were classified into putative pyramidal neurons and interneurons; the putative pyramidal neurons were identified based on the spike width (>0.35 ms) and the mean firing rate (<5 Hz) according to [15]. A putative pyramidal neuron was considered active if it fired at least 100 spikes during locomotion, and only such cells were included in the subsequent analysis.

### 2.6 Place cell analysis

All spike and positional data were speed-filtered: the data recorded during immobility (defined as monkey's running speed < 20 cm/s) were removed. Then, the linear track was divided into 10 × 10 cm bins (40 × 9 square bins). The number of spikes fired and the duration of the occupancy of each bin by the monkey were calculated. These data were individually smoothed using a Gaussian kernel with a standard deviation of σ = 1.5 bins. A 2-dimensional firing rate map was computed for each neuron by dividing bin-by-bin the spike count by the time duration. Unvisited bins (where the total time spent was <125 ms before smoothing) were discarded from analysis to prevent rate inflation due to low occupancy. For each neuron, the firing rate map for each task trial was examined to determine spatial information content. The cells, whose spatial information content exceeded the 95th percentile of the distribution constructed from appropriate surrogate data were regarded as place cells.

### 2.7 Analysis of movement-directional tuning

An instantaneous movement direction of the monkey at each location was calculated along the vector from the sequential location 33.3 ms before to the observed location. The directional data were binned into 10 bins of 36° each, and computed the firing rate in each bin by dividing the number of spikes in that bin by the time the animal spent in that bin. The peak firing rate was defined as the highest rate in the head-direction tuning curve. The directionality of the tuning curve was quantified by computing the Rayleigh vector length of the circular distribution, using the following definition [3]:

$$\frac{\pi }{n\cdot \sin\left(\frac{\pi }{n}\right)}\sum_{j=1}^{n}r_{\theta_{j}}e^{-i\theta_{j}}/\sum_{j=1}^{n}r_{\theta_{j}}$$

where *n* is the number of circular movement-direction bins, *θ*_*j*_ is the direction in radians of the *j-*th circular bin (namely, 2*π j/n*), and *r*_*θj*_ is the average firing rate for a given movement direction. Intra-trial stability was calculated by dividing the recording trial into two equal time bins and computing the Pearson product-moment correlation between spatially corresponding bins from the first and second half of a single trial, using only those bins in which firing rate >0 Hz in at least one half of the trial [4].

## Funding

This work was supported by JSPS KAKENHI Grant Numbers JPK26350992 and JP17K01991.
